# The “TSH Receptor Glo Assay” – A High-Throughput Detection System for Thyroid Stimulation

**DOI:** 10.3389/fendo.2016.00003

**Published:** 2016-01-28

**Authors:** Rauf Latif, Zerlina Lau, Pamela Cheung, Dan P. Felsenfeld, Terry F. Davies

**Affiliations:** ^1^Thyroid Research Unit, Department of Medicine, Icahn School of Medicine at Mount Sinai and the James J. Peters VA Medical Center, New York, NY, USA; ^2^Integrated Screening Core, Experimental Therapeutics Institute, Icahn School of Medicine at Mount Sinai, New York, NY, USA

**Keywords:** TSH receptor, small molecule, screening, high-throughput, thyroid-stimulating hormone

## Abstract

**Background:**

To identify novel small molecules against the TSH receptor, we developed a sensitive transcription-based luciferase high-throughput screening (HTS) system named the TSHR-Glo Assay (TSHR-Glo).

**Methods:**

This assay uses double-transfected Chinese hamster ovary cells stably expressing the human TSHR and a cAMP-response element (CRE) construct fused to an improved luciferase reporter gene.

**Results:**

The assay was highly responsive toward TSH in a dose-dependent manner with a TSH sensitivity of 10^−10^M (10 ± 1.12 μU/ml) and thyroid-stimulating antibodies, a hallmark of Graves’ disease, could also be detected. The assay was validated against the standard indicator of HTS performance – the *Z*-factor (*Z*′) – producing a score of 0.895. Using the TSHR-Glo assay, we screened 48,224 compounds from a diverse chemical library in duplicate plates at a fixed dose of 17 μM. Twenty molecules with the greatest activity out of 62 molecules that were identified by this technique were subsequently screened against the parent luciferase stable cell line in order to eliminate false positive stimulators.

**Conclusion:**

Using this approach, we were able to identify specific agonists against the TSH receptor leading to the characterization of several TSH agonist molecules. Hence, the TSHR-Glo assay was a one-step cell-based HTS assay, which was successful in the discovery of novel small molecular agonists and for the detection of stimulating antibodies to the TSH receptor.

## Introduction

The thyrotropin-stimulating hormone receptor (TSHR), expressed on the cell surface of thyrocytes, initiates the major trophic signals that direct thyroid cell growth and hormone synthesis/secretion (1, 2). The TSHR is also a target autoantigen in autoimmune thyroid disease, especially Graves’ disease (3–5). In addition to its expression on the thyroid cell, it is now well established that the TSHR is expressed in a variety of extra-thyroidal tissues, including bone cells, fibroblasts, and adipocytes, where it is known to modulate target cell function (6–11). In the thyroid, TSHR activation and coupling of G proteins leads to a cascade of complex intracellular events, resulting in thyrocyte growth and hormone production. Activation of Gαs stimulates the production of adenyl cyclase, which leads to an increase in cyclic adenosine monophosphate (cAMP) generation, whereas simultaneous Gq stimulation leads to PI3 kinase activation. The increases in cAMP and PI3 kinase *via* protein kinase A (PKA) and phospholipase C (PLC) cause the thyrocyte growth, differentiation, and thyroid hormone synthesis. In addition to engaging Gαs and Gαq, the TSHR is capable of activating the MAP kinase pathway *via* Gβγ and RhoGEF through G12/13. It is not yet clear as to which isoform of the G proteins is effectively used in the different tissues modulating the function of the TSHR. However, the use of either small molecules that bind to an orthosteric/allosteric site on the receptor or monoclonal antibodies to the receptor ectodomain have great therapeutic potential in the treatment of patients with thyroid dysfunction, especially hyperthyroid Graves’ disease, extra-thyroidal manifestations of Graves’ disease in the eyes and skin, and also in the investigation and treatment of differentiated thyroid cancer (12).

Recently, small molecular agonists and antagonists to the TSHR have become attractive therapeutic options because of their low cost of synthesis and ability to efficiently cross the plasma membrane of cells compared to recombinant TSH. These novel small molecules can be identified either by high-throughput screening (HTS) of large chemical libraries or by virtual docking and chemoinformatic approaches or a combination of both. The first small-molecule agonist against the TSHR was described nearly 8 years ago (13) and has been subjected to chemical modifications to improve its specificity and potency (14), and we have described two more recent molecules with similar potency but different structures (15). Such small-molecule agonists to the TSHR have the potential to be used as an effective substitute to recombinant TSH, which is difficult and expensive to produce (16) and, furthermore, small molecules may be more effective probes in the detection of thyroid cancer. In contrast, potent antagonists against the TSHR would be useful for the treatment of hyperthyroid Graves’ disease by blocking thyroid-stimulating antibodies without the risk of anti-thyroid drug side effects (17) and may also have a role in treating Graves’ eye disease (18). To date, it is not clear if the reported small-molecule ligands have enough potency to enter clinical trials. Hence, there remains a strong need for identifying and developing additional novel small molecules of higher potency, better stability, and improved pharmacodynamics and pharmacokinetics (19).

High-throughput screening methods are required to incorporate speed, efficiency, robust signal detection, and low reagent consumption in addition to sensitivity, reproducibility, and accuracy of detection. A standard HTS assay needs to be in a format that is easily adaptable for automated liquid handling and signal detection systems. Thus, to identify novel small-molecule agonists to the TSHR, we developed an in-house HTS assay called the TSH Receptor Glo assay (TSHR-Glo). This is a simple, one-step and cell-based functional cAMP assay, which has proven amenable for automation and liquid handling in a 348-well format for HTS of compound libraries. It is sensitive, reproducible, and cost-effective. Although this method was already used to screen small-molecule agonists to the TSH receptor (15), this paper describes in detail the full evaluation of optimization and validation of this assay.

## Materials and Methods

### Materials

Bovine TSH (Cat # T8931) and forskolin (Cat # F6886) were purchased from Sigma-Aldrich (St. Louis, MO, USA). The lysis and luciferase substrate (Bright-Glo) reagent utilizing a one-step preparative procedure was purchased from Promega Corporation, WI (Cat # E2610). Selected small molecules were purchased through ChemBridge Corporation, San Diego, CA, USA. Cell culture medium including Ham’s F12 (Cat # 10-080-CV) was purchased from Cellgro, Manassas, VA, USA and fetal bovine serum (FBS) (Cat # S11195) and calf serum (S11495) were purchased from Atlanta Biologicals, Flowery Branch, GA, USA.

### Generation of Stable Cells for Bioassay

We generated stable double transfectants by using a pGL4.29 [luc2P/CRE/Hygro] construct, having a minimal promoter driving a CREB response element tagged to a modified form of luciferase reporter gene luc2P (Promega Corporation, Madison, WI, USA), into our stable line of Chinese hamster ovary (CHO) cells expressing the human TSH receptor with a N-terminus hemagglutinin (HA) tag (CHO-HA:TSHR cells) (20). Several stable clones harboring both the TSH receptor and luciferase construct were selected by using high concentrations of hygromycin (800 μg/ml). To test for false positive hits in our screen, we also generated a stable line of parent CHO cells with the luciferase construct lacking the TSH receptor by transfecting the pGL4.29 (luc2P/CRE/Hygro) construct into the CHO cells and selecting with hygromycin. The functionality and specificity of these parent stable clones were selected based on different concentrations of forskolin and their total unresponsiveness to TSH. These stable cell lines were maintained in Ham’s F-12 medium with 10% of FBS, 100 IU/ml of penicillin, 100 μg/ml of streptomycin, and 50 μg/ml of hygromycin.

### TSHR-Glo Assay

The screening assay employed a selected high expressing stable line of CHO-HA-TSHR luciferase cells as described above. Activation of the TSH receptor by TSH or an agonist in these cells results in the release of Gsα from the trimeric G-protein complex for adenylate cyclase coupling increases intracellular cAMP, which then binds to the CRE promoter resulting in the transcription of luciferase gene and accumulation of luciferase enzyme within the activated cells. Luciferase in these cells was detected after lysing the cells using the commercial substrate Bright-Glo. Although the assay was initially developed on a standard 96-well plate, it was subsequently miniaturized to function in a 384-well format in a total assay volume of 16 μl to minimize reagent consumption and assay costs. Compounds were added from a 384-well stock plate using pin tool, thus transferring 17 nl from 10 mM stock into 10 μl of culture medium containing the cells. This single-step assay was easily adaptable to liquid handling and automation.

For HTS, we seeded 15,000 cells of HA-TSHR luci cells per well in a 384-opaque white bottom poxi-plate (PerkinElmer-ProxiPlate, Cat # 6008230) using Multidrop Combi dispenser (Thermo Fisher) in 10 μl of Ham’s F12 complete medium and incubated overnight at 37°C in a 5% CO_2_ incubator with relative humidity of >85%. Small-molecule libraries were transferred from 384-well stock plates containing 10 mM solutions in DMSO (Chembridge Corporation, San Diego, CA, USA). Small-molecule addition was accomplished using a 384-tip pin tool (V&P Scientific, San Diego, CA, USA) transferring 17 nl per pin (based on fluorimetric calibration), resulting in a final concentration of 17 μM per well. Plate validation and normalization controls, including negative control (medium only) with 0.1% DMSO and a positive agonist molecule previously described (14), were added to blank wells located in the first two and last two columns of each plate. Following compound addition, plates were incubated for 4 h at 37°C. At the end of 4 h, the cells were lysed by adding 6 μl of Bright-Glo reagent and incubated for 2 min before measuring luciferase activity using an EnVision Multilabel Plate Reader (PerkinElmer, Branford, CT, USA).

### HTS Screening

The L1 library, a collection of 100,000 structurally diverse molecules from Chembridge Corp., San Diego, CA, USA, selected based on their drug-like properties as defined by Lipinski’s “Rule of Five” (21), was obtained from the Integrated Screening Core at Icahn School of Medicine at Mount Sinai, NY, USA. The actual screen of the compound library was performed at a final concentration of 0.1% DMSO so that the final concentration of DMSO did not interfere in the assay.

### Intra- and Inter-Assay CV

To determine the inter assay variation of the TSHR-Glo bioassay, we tested our positive control at 17 μM in five different plates in triplicates and calculated the mean and SD and for the intra-assay variation, we took five calculations of the positive control at 17 μM in the same plate in duplicates and calculated the mean and SD for each of these readings and % coefficient of variability (CV) for them followed with an average% CV. The results of this are shown as tables.

The heteroscedasticity of this assay was also examined by taking different dilutions (0–10^5^ μU/ml) of bovine TSH in 15,000 cells plated in 384-well plate and performed the assay five times in same plate and similar dose–response done in five different plates at different time points. The results of this were plotted as graph and % CV for this is indicated.

### Serum Samples

Serum samples used in this study were unidentifiable stored samples originally collected with the full consent of the patients.

### Data Analyses

All curve fittings were performed using GraphPad Prism version 5.02.

## Results

### Assay Optimization

**Figure 1:**
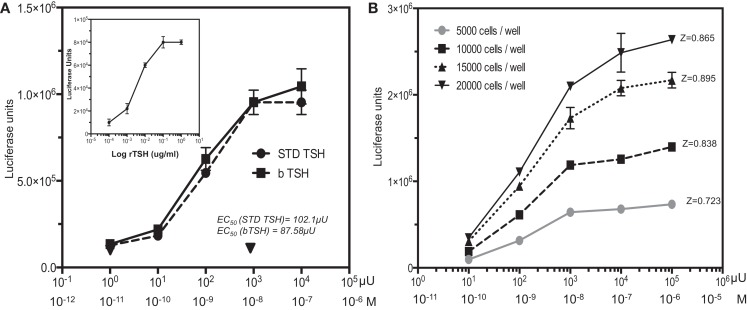
**TSH dose–response and HTS optimization**. **(A)** The TSHR-Glo cells were tested against bovine TSH and a WHO international standard human TSH (81/565 obtained from NIBSC). Inset: shows the response curve using recombinant human TSH. **(B)** To optimize the use of the assay as an HTS method, we performed a similar TSH dose–response using different cell densities on a proxy-shallow 384-well plate and calculated the *Z* factor scores under each cell density. A *Z* factor score of 0.895 with 15,000 cells/well was adopted as the standard format of cell density for HTS screening [note: 1 ≥ *z* ≥ 0.5 is considered an excellent assay (23)]. Luciferase is measured on a luminescence based microplate reader after lysis of the cells with a one-step reagent and expressed as luciferase units (LU).

To develop the reporter system, a stable clone of double-transfected HA-TSHR and CRE luciferase (HATSHR-Luci#1) cells was selected from several double-transfected clones using high concentrations of neomycin sulfate (G418) and Zeocin. We examined this clone for sensitivity to TSH in a dose–response stimulation assay with commercial bovine TSH and the WHO International human TSH standard (81/565 obtained from NIBSC), which is the same pituitary extract as the second IRP for TSH 80/558 stock. Both TSH preparations showed good correspondence with each other in the TSHR-Glo assay (Figure 1A). We then used this heterologous cell (CHO) overexpressing the TSH receptor in our HTS. The bioassay was sensitive enough to respond to 10 ± 1.12 μU/ml of TSH (10^−10^M).

In agreement with the bovine TSH and International human TSH Standard, a similar dose–response to recombinant human TSH was observed (Figure 1A inset) indicating that the activation of CRE luciferase was not due to any other contaminating proteins. CHO cells transfected with only the luciferase construct showed no responses to bovine TSH as indicated by the inverted triangle in Figure 1A. In order to scale down the assay to HTS format, we examined the effect of different cell densities ranging from 5000 cells per well to 20,000 cells per well in shallow proxy plates cultured in 10 μl of medium overnight with >85% relative humidity. We selected 15,000 cells per well for all subsequent measurements since this displayed the highest signal-to-baseline ratio after the 4-h incubation time with TSH giving a *Z* factor score of 0.895 between 0 and 10^−8^M (1000 μU/ml) of TSH (Figure 1B).

**Figure 2:**
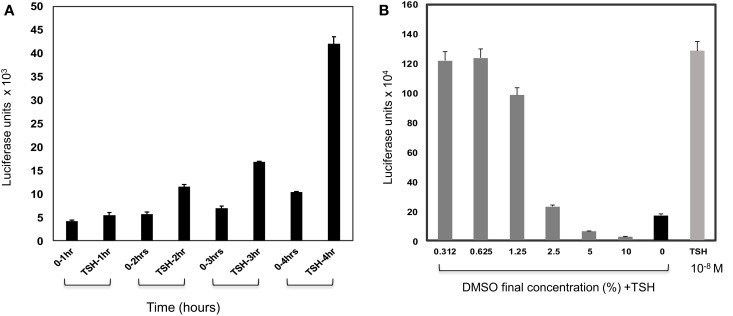
**Effect of time and DMSO on TSHR-Glo assay**. **(A)** Since this is a transcriptional based assay, we wanted to evaluate the effect of time. For this, the assay was performed with 10^−9^M (100 μU/ml) of TSH and time periods of 1, 2, 3, and 4 h without (0TSH) and with TSH treatment. Though a measurable difference in response to TSH was observed in 2 h, the response at 4 h was taken as the optimum time of incubation required for the screening because of the robust signal. **(B)** Since the library compounds were held in DMSO, it was imperative to test the effect of DMSO on the TSHR-Glo assay. The bars show the effect of increasing concentrations of DMSO on stimulation of the cells with TSH. We found that the bioassay could tolerate DMSO concentrations up to 1%. However, the concentration of DMSO in our actual screen was 0.1% DMSO.

Time-course experiments from 30 min to 4 h using 10^−9^M (100 μU/ml) of TSH indicated that a signal-to-basal ratio of >2-fold was only achieved when the incubation time reached 4 h or greater suggesting that the assay, although sensitive, was not a rapid technique (Figure 2A). An incubation time of 4 h after the addition of the compounds was chosen as the endpoint for the assay based on the difference between the signal and the background observed. Since the majority of library compounds are solubilized in dimethyl sulfoxide (DMSO), we also examined the effect of DMSO on TSH responsiveness. By this titration of DMSO, we observed a tolerance of 0.625% of DMSO, which had no detectable effect on the TSH-stimulated signal in the TSHR-Glo assay (Figure 2B).

**Figure 3:**
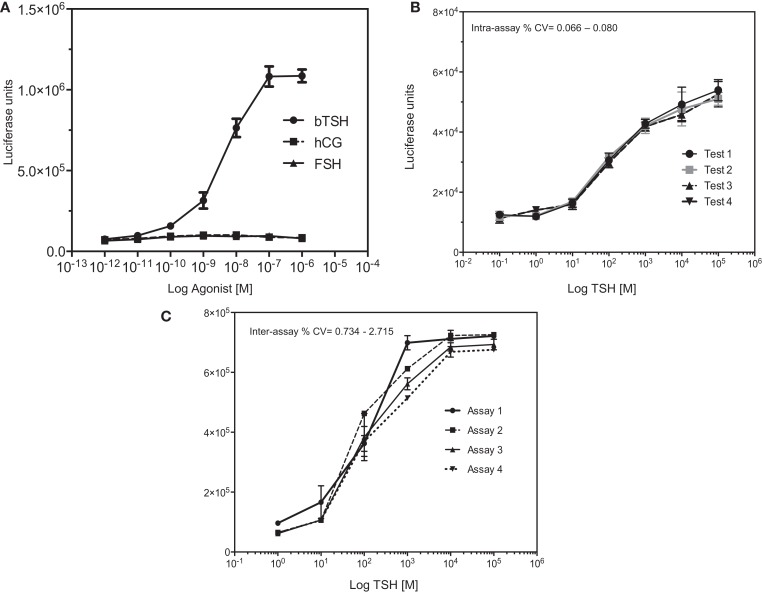
**Specificity of the TSHR-Glo assay**. **(A)** The specificity of the assay was examined by dose–responses curves against TSH, hCG, and FSH hormones. As indicated, the assay showed no responses to hCG or FSH at the concentrations tested here but a dose-dependent increase in luciferase signal. **(B,C)** In order to test the heteroscedasticity of the assay, we measured response of increasing doses of TSH in an inter- and intra-assay format as indicated by graphs. Intra-assay exhibited very minimal variability compared to the inter assay though both had CVs <5%.

The specificity of the assay was assessed by stimulating the reporter cell line with FSH and hCG ligands for homologous (GPCR) receptors, which are not expressed in this CHO cell line. As expected, neither of these hormones had any effect while increasing concentrations of bovine TSH elicited the expected response (Figure 3A).

In order to express the precision and reproducibility of the TSHR-Glo assay, we measured the inter- and intra-assay CV and gaged the heteroscedasticity in the assay (Figures 3B,C). These studies indicated a tight fit for the intra- and inter-assay variability with low CV when using a reported agonist as a positive control (14) (Tables 1 and 2). The mean inter-assay CV was 15%, whereas the intra-assay % CV was 4.38. These values indicated the suitability of the TSHR-Glo assay for further use as an HTS assay for identification of small-molecule agonists to the TSHR.

**Table 1:** **Inter-assay coefficient of variability of the TSHR-Glo assay**.

Inter-assay CV			
Control	Result (LU)	Plate	Mean
1	352,506	1	
2	280,657		
3	318,836		317,333
1	270,003	2	
2	284,091		
3	313,812		289,302
1	389,570	3	
2	390,237		
3	405,448		395,085
1	404,723	4	
2	426,760		
3	411,320		414,267
1	380,766	5	
2	381,992		
3	417,211		393,323

	Mean of mean		361,862.13
	SD of mean		54,972.04
	% CV of means		15.19

**Table 2:** **Intra-assay coefficient of variability of the TSHR-Glo assay**.

Intra-assay CV					
Control	Result	Result2	Mean	SD	% CV
1	352,506	354,343	353,424.5	1298.955157	0.367533987
2	280,657	291,041	285,849	7342.596816	2.568697745
3	318,836	304,639	311,737.5	10,038.79497	3.220271855
4	284,091	330,204	307,147.5	32,606.815	10.61601185
5	313,812	291,778	302,795	15,580.39082	5.145524469

			Average% CV		4.38

### TSH Receptor Antibody Responses

**Figure 4:**
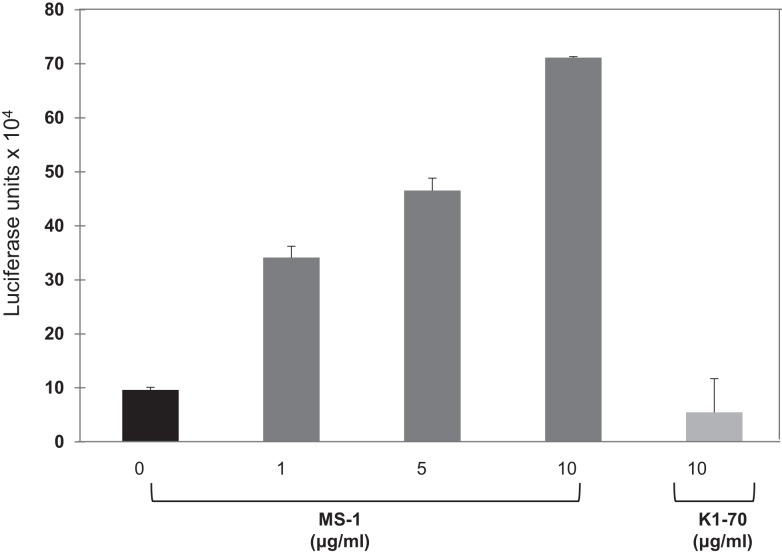
**Response of TSHR-Glo against stimulating and blocking monoclonal antibody – further proof of the specificity of the assay was obtained by testing a stimulatory monoclonal antibody (MS-1) and blocking (non-stimulatory) monoclonal TSHR antibody (K1-70) in the assay**. MS-1 stimulated luciferase activity in a dose-dependent manner, whereas K1-70 was unable to show any response in the assay even at the highest concentration.

**Figure 5:**
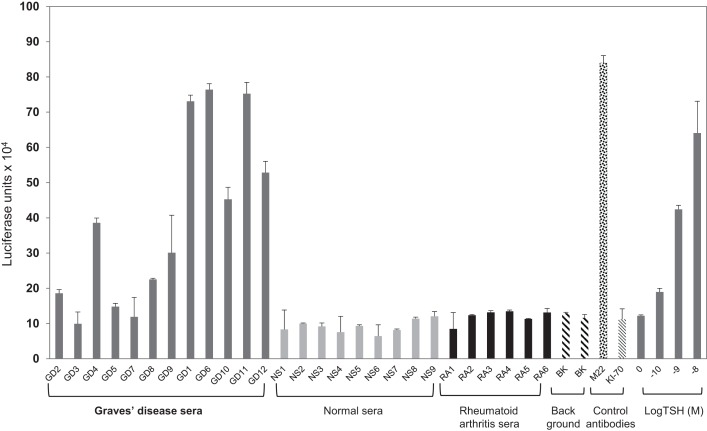
**Agonist activity measured in patient sera using TSHR-Glo**. Graves’ disease patient and normal sera were diluted 1:5 in medium. Ten of the 12 GD (Graves’ disease) serum samples (denoted GD with dark gray bars) stimulated the production of cAMP in contrast to no stimulation observed with any of the normal (denoted NS with light gray bars) or rheumatoid arthritis (denoted RA with black bars) patient sera. Background is denoted by the two filled hatched bars, a human-stimulating monoclonal antibody (M22 shown as hatched bar), human-blocking monoclonal antibody (K1-70 shown as filled criss-cross bar) 1 μg/ml, and bovine TSH (gray bars) at increasing log concentrations.

We initially observed that a monoclonal thyroid-stimulating antibody induced potent responses in the system, while a TSH receptor-blocking monoclonal antibody (22) was incapable of signaling (Figure 4). To determine if the TSHR-Glo assay was useful in measuring stimulating antibodies in patients with Graves’ disease, we used sera with known TSHR antibodies and control sera that were negative (Figure 5). The data indicated that the TSHR-Glo assay provides a sensitive (limits of detection: 10–100 mIU/l) and a selective assay for also measuring agonist-like responses in Graves’ disease patient’s sera.

### High-Throughput Screening of a Chemical Library

**Figure 6:**
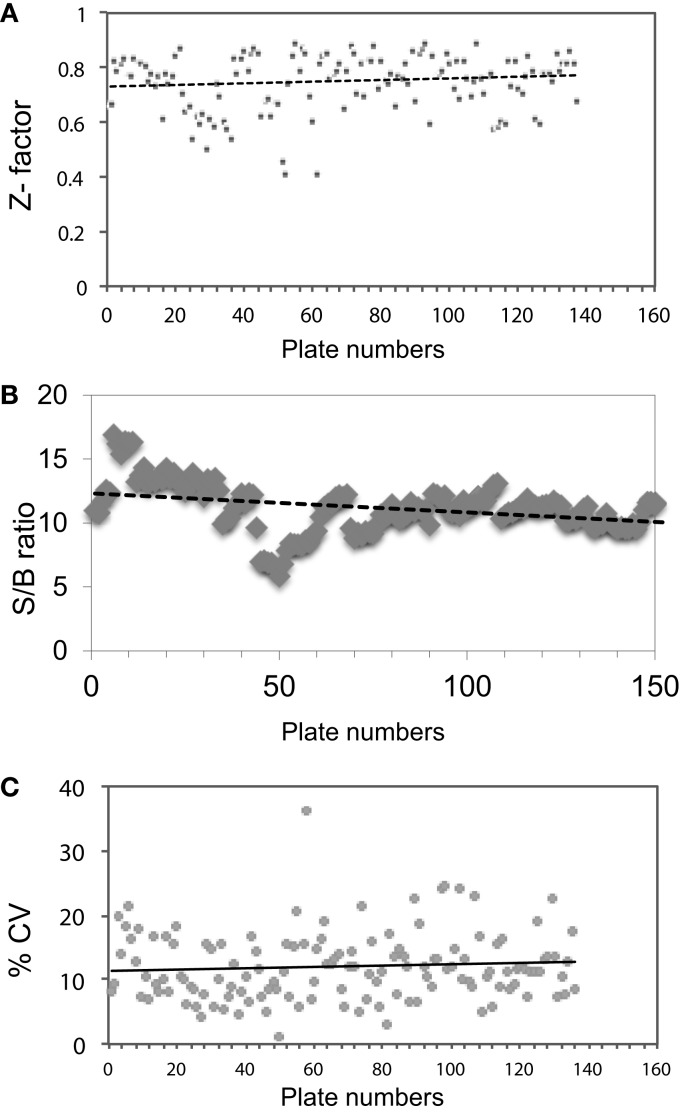
**Evaluation of HTS performance**. **(A)** *Z* factor was calculated by taking the positive control and basal control responses of each duplicate plate and using the formula *Z*′ = 1 − (3 × SD of total signal + 3 × SD of basal signal)/(total signal − basal signal) as indicated by Zhang et al. (23). **(B)** Signal-to-background ratio (S/B): total signal was obtained from alternating wells of columns 1 and column 22 using 17 μM of positive control and background signal was were collected from the alternating wells of column 1 and column 22 treated with medium containing 0.1% DMSO. **(C)** Coefficient of variation (% CV), calculated as the SD from the wells in the basal medium divided by the wells of the positive control in all 137 plates.

Screening a total of 137 plates with 384 wells each using the TSHR-Glo assay, we obtained a *Z*-factor in the range of 0.7–0.8 based against a positive control used on each plate (Figure 6A). These *Z* values exceeded the commonly accepted threshold (0.5) for validation of high-throughput assays (23). The signal-to-background ratio was linear and the mean CV was within an acceptable value 5–20% for a bioassay (Figures 6B,C). Using this HTS assay, we examined 48,224 molecules for agonist activity before suspending the screen because of the large number of positive results (hits) based on our selection criteria. All of the compounds were screened at a single concentration of 17 μM in duplicate plates. We then selected positive samples only if a “significant” response was obtained in both plates, with significance defined as a response greater than mean ± 3 SD compared to the negative control. This screening resulted in 63 responder molecules yielding a hit value of 0.13%. We subjected 21 of the molecules that generated the strongest signal to a second confirmatory testing using CHO luciferase cells (parent CHO cells without the TSH receptor but stably transfected with luciferase construct). Based on this second round of testing for false activation, we eliminated two molecules that stimulated the non-TSHR containing CHO cells. We then selected two compounds, named MS437 and MS438 (marked with an asterisk), as our lead molecules, which showed >10-fold responses above the baseline and no activity on the parent CHO luciferase cells. These lead molecules were further characterized for their specificity, binding, and their *in vivo* activity as described elsewhere (15).

## Discussion

The thyroid-stimulating hormone receptor is not only a major regulator of thyroid function but also plays a major role in several thyroid pathologies, including hyperthyroidism, hypothyroidism, and thyroid tumors (5). Further, its ubiquitous presence in other tissues of the body, such as adipocytes, fibroblasts, bone cells, and immune cells, has expanded its role in these tissue depots (24), although the physiological relevance of the receptor in these extra-thyroidal sites is still being revealed. Though various effective treatment strategies are currently available for the treatment of thyroid conditions, not all of these are devoid of side effects (25). Thus, there has been great impetus to develop newer therapeutics for the treatment of thyroid disease, especially Graves’ disease. One such therapeutic area of development has been identifying small molecules, which can bind to the receptor and modify their signaling in a positive or negative allosteric effect (19). In this manuscript, we describe in detail the development and use of a high-throughput assay for identifying novel small-molecule agonists against the TSH receptor.

Since the TSH receptor is known to couple to various G proteins (26), bioassays have been largely designed to measure their activation. Fluorescence polarization, homogenous time-resolved fluorescence (HTRF) or enzyme fragmentation complementation, and cyclic nucleotide-gated ion channel (CNG) – coupled are some of the commonly used approaches to measure Gs activation in GPCRs. One such CNG-coupled cAMP assay employing a membrane potential dye has been previously described as a high-throughput assay for screening agonists against the TSHR (27). Auto-fluorescing compounds constitute a large bulk of compound libraries, thus discouraging us from using such a fluorescence-based readout for bulk screening and providing an advantage to a transcriptional-based luciferase cAMP assay described here. The TSHR-Glo assay was based on a stable overexpressed system; it is robust in producing signal and easily meets the criteria for an HTS assay by having high *Z* scores. In addition, over-expressed systems are well known to produce a better signal-to-noise ratio than native lines. Furthermore, CHO cells, in particular, are easy to culture and robust enough to be handled by automation. The TSHR-Glo assay described is based on double-transfected CHO cells harboring the full-length receptor and CRE response element tagged to an improved synthetically derived luciferase reporter gene (luc2P). Luc2P is a modified firefly luciferase sequence with humanized codon optimization that is designed for high expression and reduced anomalous transcription. In addition, the luc2P gene contains hPEST, a protein destabilization sequence, which further reduces background transcribed protein (28). All these factors had a cumulative effect in developing this assay for HTS of a 50k chemical library. The positive hits were initially confirmed for specificity using the parent cells without the receptor but having the CRE luciferase vector stably transfected into them. These cells responded well to forskolin, an adenylyl cyclase activator but not to TSH. Further confirmation of the specific hits was obtained with other orthogonal assays, such as real-time PCR in native cells and with *in vivo* testing (15).

Although the TSHR-Glo assay is a sensitive and robust HTS assay, it is not a rapid technique because the time between the addition of stimulant and final read is 4 h. We think that the time lag in the assay is due to the time-dependent accumulation and folding of luciferase within the cells needed to reach a detectable threshold unlike other enzyme-linked assays that directly measure second messengers, such as cAMP. As an HTS assay, the TSHR-Glo assay also showed a good tolerance to DMSO concentrations within the working limits of any library screening. Further, on checking the specificity of this assay against FSH and hCG responses, we found almost no response. The assay also had good intra- and inter-assay% CV as indicated in Figure 4 and Tables 1 and 2.

To identify hits from large chemical libraries, an assay has to be sensitive and precise, and thus, a successful screen depends critically on the quality of the HTS assay used. Although the criteria or parameters for evaluating the suitability of an HTS assay for hit identification are not well defined, a screening window coefficient, called the *Z*-factor is accepted as the “gold standard” (23). *Z*-factor is a dimensionless, simple statistical coefficient, which is reflective of both the assay signal dynamic range and the data variation associated with the signal measurements, and therefore, it is suitable for assay quality assessment. The *Z*-factor has proven to be a useful tool in HTS assay optimization and validation (23). TSHR-Glo assay proved to have an excellent *Z* score of 0.895 to fit this criteria and which was well above the *Z* factor score of >0.5 considered as a satisfactory standard for a HTS assay (23). The consistency of these parameters throughout the screen is reflected (Figure 6). The TSHR-Glo bioassay responded to 10 μU/ml of TSH (10^−10^M) as found in many other sensitive TSH bioassays (26, 29).

Graves’ disease is marked by the production of different autoantibodies directed against the ectodomain of the TSHR. The characteristic hyperthyroidism in Graves’ disease is due to the presence of stimulating antibodies although blocking and cleavage region (or neutral) antibodies to the TSHR are also produced in many patients. The only way to distinguish these different types of antibodies is by their differential signaling ability. By using a hamster thyroid-stimulating monoclonal antibody (MS1) derived in our laboratory (30) and a human blocking antibody (22), we could ascertain that the TSHR-Glo assay was able to distinguish between a stimulator and blocking antibody. Further, this assay was capable of detecting thyroid-stimulating antibody positive sera versus normal controls and other diseases (Figure 6).

In summary, we describe a simple HTS assay (TSHR-Glo) for identifying agonist molecules that activate the TSH receptor. This assay has the potential to be used for (1) screening chemical libraries for the identification of lead agonists to the TSH receptor, (2) an assay for thyroid-stimulating antibodies of Graves’ disease, and (3) for the selection of monoclonal antibodies against the TSH receptor.

## Author Contributions

RL – developed the assay and optimization and did the experiments, analyzed the data, and wrote the manuscript. ZL, PC, and DF – did the screening of the chemical library. TD – data analysis and manuscript preparation.

## Conflict of Interest Statement

The authors declare that the research was conducted in the absence of any commercial or financial relationships that could be construed as a potential conflict of interest.

## Abbreviations

CHO, Chinese hamster ovary cells; HTS, high-throughput screening; SML, small-molecule ligands; TSH, thyroid-stimulating hormone; TSHR, TSH receptor; TSHR-Glo, TSH receptor glow.
